# What is the Zanzibari craftswomen’s experience on eyeglass use, business challenges and solutions? Participatory action research using solicited diaries and listening workshop narratives

**DOI:** 10.1136/bmjopen-2024-090883

**Published:** 2025-06-10

**Authors:** Fatma Omar, Bhagyalaxmi Shivalingam Pillai, Omar Juma Othman, Eleanor Holland, Eden Mashayo, Kajal Shah, Ronnie Graham, Christine Graham, Ving Fai Chan

**Affiliations:** 1 Zanzibar Ministry of Health, Zanzibar, United Republic of Tanzania; 2 Centre for Public Health, Queen’s University Belfast, Faculty of Medicine Health and Life Sciences, Belfast, UK; 3 School of Medicine, Dentistry and Biomendical Sciences, Queen’s University Belfast, Faculty of Medicine Health and Life Sciences, Belfast, UK; 4 Vision Care Foundation, Dar-es-Salaam, United Republic of Tanzania; 5 Dublin Institute of Technology, Dublin, Ireland; 6 Independent Researcher, Edinburgh, UK

**Keywords:** Community-Based Participatory Research, PUBLIC HEALTH, QUALITATIVE RESEARCH

## Abstract

**Abstract:**

**Objective:**

To present older presbyopic Zanzibari craftswomen’s firsthand experiences with the eyeglasses, the challenges they face in advancing their businesses and proposed solutions to these challenges.

**Design:**

This participatory action research used solicited diaries, where 10 craftswomen documented their experiences with presbyopia correction for 6 months. The diaries were translated into English for qualitative content analysis. Researchers then held a 2-day listening workshop with 20 craftswomen to discuss the diary findings and gather their perspectives on challenges and solutions. These workshops included group discussions and debates to encourage open communication. Narrative analysis was conducted to identify the key narratives.

**Setting:**

Zanzibar, Unguja and Pemba Islands.

**Participants:**

Zanzibari craftswomen entrepreneurs with corrected presbyopia, 40 years and older.

**Intervention:**

Presbyopia near vision eyeglasses for 6 months.

**Results:**

The study found that improved vision with eyeglasses significantly benefits craftswomen in Zanzibar. They experience increased work efficiency, quality and income. Craftswomen also reported greater independence, confidence and participation in daily activities. However, limited market access and competition restrict their income growth. Business skills training in areas like marketing and finance is seen as a solution for sustainable success.

**Conclusions:**

Improved near vision was associated with enhanced productivity, financial confidence and well-being among craftswomen. However, persistent barriers—including limited market access, competition and lack of business skills—highlight that a vision-only approach may not address the intersectional challenges faced by older women entrepreneurs in Zanzibar. These findings suggest that integrated strategies combining vision care with business mentoring may offer more sustainable support.

**Strengths and limitations of this study**

- Solicited diaries and listening workshops offer a powerful combination of real-time personal reflection and collective dialogue, capturing both the nuanced, lived experiences and evolving impact of vision correction on individuals’ daily lives.
- Solicited diaries may not fully capture the broader social and cultural context influencing participants’ experiences.
- Participants may have under-reported negative experiences due to social desirability or self-censorship.
- Narrative data are subjective and may not be generalisable beyond the study population.
- Findings reflect the experiences of older craftswomen in Zanzibar and may not apply to other contexts or age groups.

## Introduction

 Presbyopia, a common, age-related condition that affects the ability to see clearly at close distances, hampers near-vision tasks. In 2019, low-income and middle-income countries (LMICs) had 238.4 million individuals with uncorrected or undercorrected presbyopia, causing potential productivity losses of US$54.13 billion.^1^ Females are disproportionately affected,^2^ constituting 56% of the blind and 55% of those with vision impairment globally.^3^ Despite affordable eyeglasses leading to positive outcomes,^4^ a review found only three eye health programmes targeting women in LMICs,^5^,^7^ all showing positive outcomes such as increased eye health knowledge, better practices, enhanced confidence, improved productivity and quality of life.

**Figure 1:**
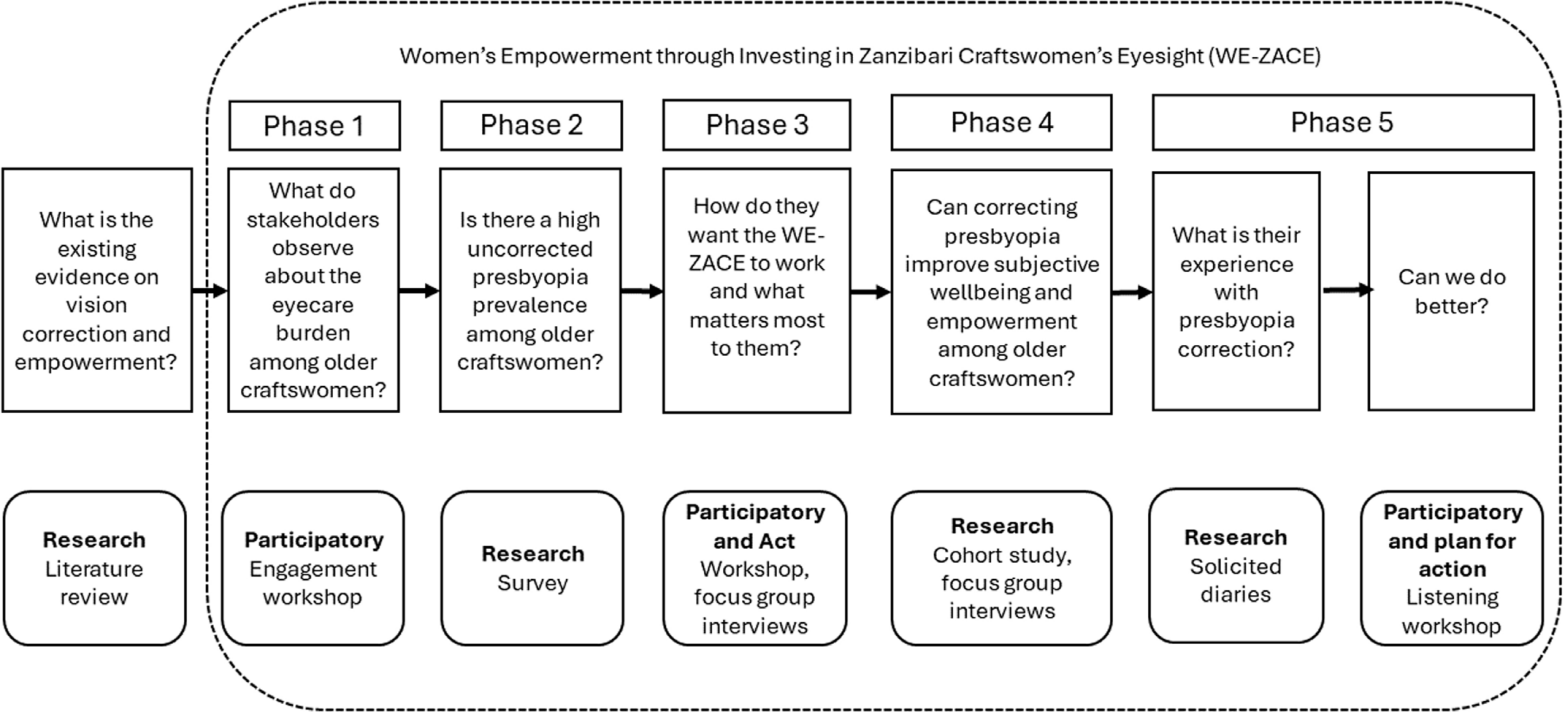
The five phases of the Women’s Empowerment through Investing in Zanzibari Craftswomen’s Eyesight participatory action research.

In 2012, presbyopia prevalence in Zanzibar was 89.2%, with only 17.6% eyeglasses coverage due to financial and prioritisation barriers.^8^ Interventions achieved a 93.6% eyeglasses retention rate and 89.5% satisfaction.^9^ Older craftswomen entrepreneurs in Zanzibar, whose tasks involve beading, tailoring and pottery, require precise vision and face significant eye health issues that impact their productivity and financial stability. Following discussions with Zanzibar’s Ministry of Health, the participatory action research (PAR) Women’s Empowerment through Investing in Zanzibari Craftswomen’s Eyesight (WE-ZACE) was launched from October 2021 to May 2023 to understand the social issues faced by older craftswomen and determine how vision correction could empower them, as well as identify other interventions needed to promote social change. The WE-ZACE project included five interconnected phases, with craftswomen participating in each phase (PAR) (figure 1). PAR is a collaborative approach that involves participants as coresearchers throughout the research process.^10^ This paper presents findings from Phase 5 of the WE-ZACE project.

### Phase 1: engagement and understanding women’s empowerment

A 2-day consultation workshop in Zanzibar^11^ with 18 local stakeholders revealed that many craftswomen experience eye irritation and need near eyeglasses. However, they often do not seek treatment due to inaccessible and unaffordable eye services, and a lack of eye health knowledge and practices. When asked about empowerment, the craftswomen identified economic, social, psychological, educational and political empowerment as the most important aspects.^11^

### Phase 2: validating burden and understanding attitudes to near vision correction

A cross-sectional survey of 263 craftswomen aged 35 and older confirmed an 86.6% prevalence of presbyopia, with only one case adequately corrected.^12^ Additionally, 29.7% had distance vision impairment, with 65.4% needing distance vision eyeglasses, none of whom were corrected.^12^ Given that social norms can influence the acceptance of glasses, we assessed their attitudes towards wearing them. Both the survey and qualitative interviews revealed positive attitudes towards glasses wearing.^11^,^13^

### Phase 3: understanding vision and women’s empowerment, developing the theory of change and designing the WE-ZACE programme

With no consensus on measuring women’s empowerment in our population, we interviewed 24 older craftswomen to understand how they perceive improved vision could empower them. They believe corrected vision could enhance income, savings, self-confidence, decision-making, social participation, leadership, skills acquisition and education.^13^ Using these insights and phase 1 findings, craftswomen developed the Theory of Change map for the WE-ZACE project.^11^ The premise is that investing in women-targeted eye health programmes, training, education and market expansion will enhance various aspects of their lives. The team then collaborated with craftswomen to develop the implementation plan and an electronic data collection tool^14^ for the project.

### Phase 4: provision of presbyopic correction and follow-up

After providing presbyopic correction to 289 craftswomen, they showed immediate improvements in subjective well-being and self-reported productivity. Over 6 months, their well-being, empowerment and financial standing were quantitatively assessed, followed by a cost–outcome analysis. We also conducted focus group discussions with craftswomen, their spouses and community leaders to understand significant changes and how men could contribute to the empowerment process. These findings will be published separately.

### Phase 5: reflection and listening workshop

The aim of solicited diaries^15^ from craftswomen is to capture their experiences with glasses by allowing them to express their feelings, thoughts and daily experiences in their own words and at their own pace. These diaries provide naturalistic data on how glasses impact various aspects of their lives, leading to authentic and insightful reflections. The longitudinal perspective helps researchers understand the long-term impact of wearing glasses. These insights were used as a basis for discussions at the listening workshop^16^ to understand challenges and find solutions. The workshop provided direct feedback, empowered participants and fostered ownership in problem-solving, offering detailed insights through guided discussions to help identify challenges and brainstorm practical, adaptable interventions.

## Methods

### Patient and public involvement

Public and community stakeholders were actively involved throughout the WE-ZACE study, from defining priority outcomes to shaping the intervention and informing data collection. Older Zanzibari craftswomen, cooperative managers, community leaders and male counterparts contributed to the codesign of the WE-ZACE intervention, advised on appropriate timing and delivery and participated in planning the evaluation process. Their input ensured that the research addressed locally meaningful needs, was delivered in trusted community settings, and respected seasonal and social contexts. This end-to-end involvement strengthened the study’s relevance, acceptability and impact, and reflects best practice in participatory research. The research is disseminated to the study participants.

### Study design and sample

This PAR used solicited diaries over 6 months to collect unfiltered, personal observations. PAR is an approach that combines systematic inquiry with action, involving those most affected by the issue under study as coresearchers. As Chevalier and Buckles^10^ describe, PAR promotes shared learning, reflection and problem-solving by valuing the lived experiences and insights of participants. Rather than treating individuals as passive subjects, PAR encourages collaborative engagement throughout the research cycle to generate locally relevant knowledge and solutions.

This approach is especially well suited to research in LMICs, where power imbalances and externally imposed interventions often undermine sustainability and impact. Involving Zanzibari craftswomen as active participants enabled the research to reflect their real-world challenges and priorities, particularly around presbyopia and economic empowerment. PAR’s emphasis on dialogue, cocreation and social change ensured that the findings were not only contextually grounded but also actionable, supporting the women in identifying barriers and generating practical strategies for their own development.

To ensure quality in this PAR study, we prioritised authentic participant involvement, transparency in decision-making and ongoing reflexivity.^10^ Craftswomen were engaged throughout the process—from identifying challenges to codeveloping solutions—enhancing the credibility and relevance of the findings. We used multiple data sources (diaries and workshops), shared interpretations with participants for validation and maintained detailed analytic records to support reflexive analysis. These strategies align with PAR quality principles, where the rigour lies not only in methodological soundness but also in the research’s ability to foster empowerment and meaningful social change.^17 18^

This PAR used solicited diaries over 6 months to collect unfiltered, personal observations. Solicited diaries were used as a reflective, participant-led method to gather first-person accounts of craftswomen’s experiences with presbyopia correction over a 6-month period, aiming to minimise research and observer bias.^15^ This approach enabled women to document their thoughts, feelings and daily challenges in a private and flexible manner,^19^ using either Kiswahili or English. Solicited diaries are particularly valuable in LMIC settings where gender dynamics or power imbalances might inhibit open verbal expression.^19^ By giving participants control over the timing, frequency and content of their entries, diaries encouraged honest, in-depth reflection while reducing potential researcher influence.^19^ The method also offered a longitudinal lens to observe change over time,^20^ particularly in relation to vision, productivity and well-being.

Diarists were able to privately share their experiences and feelings regarding the impact of presbyopia corrections on their lives, aiming to minimise research and observer bias.^15^ The 6-month duration was chosen to capture enduring changes in the craftswomen’s lives while managing participant burden and reducing attrition rates.^21^ The study focused on ten diarists from eight women’s cooperatives representing different craft types across Zanzibar’s Unguja and Pemba Islands, each with distinct socioeconomic, historical and cultural contexts. Craftswomen were recruited through their managers, with 272 women aged 40 and older invited for free eye examinations to identify those with presbyopia and proficient writing skills. 99 met the criteria. One woman from each cooperative was initially selected (n=10); however, in tailoring and weaving cooperatives with larger memberships, two were chosen to enhance diversity. Selection was based on presbyopia diagnosis and writing proficiency (table 1).

**Table 1:** Sampling frame of participants in solicited diaries and listening workshop

Location	Unguja Island				Pemba Island			
Types of crafts	Tailoring	Weaving	Pottery	Soap manufacturing	Tailoring	Weaving	Pottery	Soap manufacturing
Diarists (n=10)	2	1	1	1	1	2	1	1
Listening workshop (n=20)	3	2	3	2	3	3	2	2

Two qualitative researchers (FO, public health specialist, female and OJO, public health specialist, male) introduced themselves to selected diarists, instructing them on completing diary entries in English or Kiswahili. Over 6 months, diarists recorded thoughts, feelings and experiences related to wearing eyeglasses, employment and empowerment, encouraged to write as many entries as desired with a minimum of one per month (totalling at least six entries). Monthly follow-ups prompted reflection on various topics including work environment, family and community relationships, daily activities, and social interactions. Completed diaries were collected after 6 months, stored securely online and translated into English for analysis. Analysts (BSP, global health, female and EH, medical student, female) coded transcripts independently, maintaining detailed records for reflexivity and consensus discussions. Qualitative content analysis involved generating codes, harmonising interpretations in consensus meetings to establish a unified coding framework and themes. A third analyst (VFC, global health optometrist, male) refined themes, and participant quotes, anonymised for privacy, were used to illustrate findings (eg, Entry J1). 24 excerpts were selected for their thematic relevance and illustrative depth (online supplemental table S1). All 49 entries are available on request as they contained intimate, confidential and identifiable data.

### Listening workshop

1 month after analysing the diaries, we conducted a 2-day listening workshop with 20 craftswomen (table 1). Listening workshops were held as facilitated group discussions^22^ designed to validate diary findings, promote shared learning and codevelop solutions to the challenges identified. Conducted after diary analysis, the workshops brought together a broader group of craftswomen to reflect on emerging themes and generate collective insights. This method aligns with the PAR framework by fostering inclusive dialogue, mutual respect and community-driven action. Listening workshops are especially effective in LMIC contexts where local knowledge, group consensus and oral storytelling are culturally significant modes of engagement.^22^ They also served as a platform for empowerment, allowing participants to move from narrating personal experiences to critically analysing them and contributing to solution-focused planning.

Participants in the listening workshops (n=20) were purposively selected from the wider group of women who received eyeglasses, ensuring representation across age, geographical location and craft type. The workshop was facilitated by FO and OJO. Each session (total of four) included an icebreaker (15 min), framing activity (10 min), guiding questions (5 min), small group discussions (30 min), reporting back (20 min), break (20 min), debate (60 min), another break (60 min) and debriefing (30 min). The icebreaker involved introductions and cooperative affiliations. Framing activities clarified workshop goals, emphasising facilitators’ roles in listening to attendees’ needs, concerns, questions and comments. Guiding questions were discussed before small group discussions (4–5 women per group).

- Session 1: Corrected vision with eyeglasses improved my life. How much do you agree? Why?
- Session 2: Corrected vision with eyeglasses would be enough to empower my life. How much do you agree? Why?
- Session 3: What do you think is stopping you from achieving success in your life given your vision is now corrected?
- Session 4: How do you think these challenges could be overcome?

Facilitators ensured all participants had opportunities to speak and encouraged continuation of off-topic discussions in future sessions. A group leader summarised discussion points on a flipchart, while prompts during a 20 min break facilitated spontaneous open group discussions to explore diverse viewpoints and foster consensus among craftswomen. Three notetakers took notes in real time. During the 1-hour break, FO and OJO conducted rapid qualitative analysis of discussion notes, creating idea maps for swift data collection and interpretation. Due to time constraints, they summarised directly from notes rather than producing transcripts, maintaining study rigour. VFC and EM reviewed maps to identify key narratives through narrative analysis.^23^ Narrative analysis was employed to explore how participants made sense of their lived experiences with presbyopia correction, economic activity and empowerment. We drew on Sparkes’ (2005) approach to narrative analysis, which emphasises how personal stories reveal deeper social meanings and how individuals use storytelling to make sense of their identities and experiences.^24^ We treated diary entries and workshop discussions as rich, meaning-laden texts, focusing on the structure, content and context of the stories told. Particular attention was paid to expressions of change, identity, agency and relationships over time.

The analysis was informed by Riessman’s framework,^25^ which focused on how participants structured their experiences, expressed feelings and shifts over time and coconstructed meaning through storytelling. Our approach combined thematic and hermeneutic elements, attending both to the content of the stories and the social contexts in which they were shared. This involved moving between parts of the narrative (eg, individual diary entries or quotes) and the whole story (eg, emerging themes or shared experiences across participants) to deepen understanding. A debriefing session followed to ensure accurate reflection of craftswomen’s opinions and address any needed clarifications or changes.

## Results

### Findings from the solicited diaries

The study obtained a diarist response rate of 81.7%, comprising 49 entries. The data analysis resulted in the identification of two themes and six subthemes. The accompanying diary excerpts and diary identification numbers are shown in online supplemental table S1.

#### Theme 1: work-related benefits

##### Subtheme 1

Presbyopic eyeglasses improved near vision for craftswomen, enhancing precision, clarity and reducing eye strain.

The craftswomen observed a significant enhancement in their near vision after receiving eyeglasses that specifically catered to their needs, enabling them to perceive items with greater precision and clarity up close. The eyeglasses also notably alleviated eye strain and discomfort (entries 1 and 2).

##### Subtheme 2

Eyeglasses improved craftswomen’s work efficiency, quality and income by addressing vision issues, enhancing savings and reducing rejections.

Uncorrected vision posed challenges for craftswomen, affecting their performance in arts-related tasks such as pottery, weaving and needlework (entries 3 and 4). Despite their skills, poor vision hindered productivity and quality (entry 5). Close-distance vision issues further complicated intricate tasks, sometimes requiring family assistance (entries 6 and 7). Introduction of eyeglasses significantly improved their vision, enhancing comfort and efficiency (entry 8). This improved vision enabled craftswomen to work more proficiently, complete tasks faster, improve handiwork quality and experience greater job satisfaction (entry 9). Craftswomen reported improved work quality, leading to increased income and savings compared with previous months, with no product rejections noted (entry 10).

### Theme 2: outside-of-work benefits

#### Subtheme 1

Eyeglasses improved craftswomen’s daily activities, studies and engagement, facilitating easier participation in tasks, outings and family time.

Outside of work, the craftswomen’s presbyopia hindered their participation in various activities, including daily tasks and outings (entry 11). Moreover, prior to wearing eyeglasses, the craftswomen faced challenges with their studies and engagement in study forums (entry 12). Craftswomen experienced many benefits from wearing eyeglasses, including being able to read, write and use their phone with more ease (entry 13). They could spend more time with family outside the home and pursue other interests such as reading in the madrasa (entry 14). Additionally, the eyeglasses significantly helped them with a variety of chores, such as picking rice, homework and clipping their nails (entry 15).

#### Subtheme 2

Eyeglasses bring renewed independence, confidence to craftswomen, enabling task management, easing personal matters, boosting productivity and enhancing peer support.

Craftswomen described a renewed sense of independence and confidence after using eyeglasses. Previously difficult or inaccessible tasks become more manageable. Craftswomen are better able to carry out a variety of tasks on their own, without the need for assistance or close supervision. They are now capable of handling their own concerns, whether it be reading, taking care of their finances, or engaging in personal interests (entries 16 and 17). Additionally, the improved vision gives craftswomen greater confidence in their skills. Their increased self-assurance not only increases production but also motivates them to help their peers (entry 18).

#### Subtheme 3

Improved vision via eyeglasses enhances craftswomen’s roles as advisors and artisans, fostering positive relationships in personal and professional spheres.

Improved vision from wearing eyeglasses has enabled craftswomen to build strong connections with fellow craftswomen and members of her community. Noticing the positive impact on her vision, people in the town have sought their assistance in acquiring eyeglasses for their own eye issues. Additionally, their fellow artisans have approached her for help in improving their creations. The benefits of wearing eyeglasses extend beyond their professional life, as they have strengthened her relationships with family and friends (entries 19 and 20).

#### Subtheme 4

Craftswomen actively promote the use of eyeglasses, stressing the importance of awareness and urging vision evaluations, especially for the elderly.

Craftswomen had limited awareness and knowledge about how to address their eye condition before acquiring eyeglasses. There was a prevailing belief that their vision issues were insurmountable, leading to a reluctance to seek solutions (entry 21). Craftswomen also emphasised that a lack of awareness campaigns contributed to individuals not seeking help for their vision issues (entry 22). However, the positive vision outcome motivated them to advocate for the use of eyeglasses for vision correction, even among the elderly, urging those who have not been fitted with eyeglasses to seek evaluation for vision improvement (entries 23 and 24).

### Narrative analysis from the listening workshop

#### Narrative 1: the paradox of increased productivity and limited income

While the craftswomen, like the diarists, acknowledge the overall positive impact of glasses on their lives, there is an interesting disconnect regarding income. This difference stems from the unique challenges they face as entrepreneurs in Zanzibar.

Many craftswomen are self-employed. While improved vision leads to higher production, it does not necessarily translate to higher income. This is because they lacked the know-how to sell their products outside Zanzibar. The limited market access creates intense competition among the craftswomen. To attract customers, they often engage in price wars, driving down prices. Lowering prices often necessitates using cheaper, lower-quality materials. This is not only because they are readily available but also because good quality materials are difficult to find. Obtaining good materials often involves long-distance travel, which is expensive and time-consuming. The limited availability and high cost of good materials restrict the types of products the craftswomen can create. Designs often become simpler, potentially less appealing to tourists who might be a lucrative market segment.

#### Narrative 2: the challenge of unequal support

The competitive landscape becomes even more challenging when the craftswomen’s prices have to compete with entrepreneurs backed by international Non-Governmental Organisations (NGOs). These charities often provide significant advantages to their supported businesses, creating an uneven playing field. NGOs often buy good quality materials in bulk and offer them to their supported entrepreneurs at heavily subsidised rates or even tax-free. This gives these businesses a significant cost advantage. The supported entrepreneurs are often younger and equipped with the latest tools and equipment. This allows them to produce faster and in larger quantities, further driving down their production costs. The business plans developed by the NGO provide banks with greater confidence in the viability of these ventures. This translates into easier access to loans for expansion, allowing these businesses to grow even faster.

#### Narrative 3: the need for business skills training

While access to glasses has been a game-changer for these craftswomen, a new hurdle has emerged: a lack of business knowledge. Reading the loan application form is just the first step. To secure funding, they need to craft a business plan, a foreign concept. Additionally, they recognise their products lack polish compared with competitors—branding, design and product differentiation are areas they crave guidance in. The digital age also presents challenges, as some struggle to leverage new technologies. Perhaps the most pressing issue is internal—managing unequal effort within their teams. Training in these areas is crucial to unlock their full potential and ensure the long-term success of their businesses.

#### Narrative 4: a vision for growth

The craftswomen understand that good vision is just the beginning. To truly thrive, they propose a systematic business mentoring programme. This programme would empower them with the tools they need to excel, encompassing essential skills like financial management, bookkeeping and basic marketing strategies, brainstorming innovative product designs, fostering a spirit of experimentation and differentiation, emphasise maintaining high product quality and building strong customer relationships through attentive follow-up care.

Improving financial literacy and access to capital came up prominently. The curriculum would cover securing microloans and navigating the loan application process to acquire larger loans with favourable interest rates. This will enable them to invest in better materials and equipment, fuelling further growth.

Finally, they would like the mentorship to equip them with crucial marketing skills. For instance, how to partner with agents who can market their products beyond their village and even beyond Zanzibar, opening up new customer bases, explore the possibility of establishing a dedicated location to sell their products collaboratively, replacing individual stalls and creating a stronger market presence, and mentoring would equip younger members of their community with the skills to effectively advertise their products on social media platforms, leveraging the power of digital marketing.

## Discussion

This study used solicited diaries which showed that improved vision, financial security and well-being for craftswomen. Subsequent engagement with the craftswomen suggests that limited market access and competition restrict their income growth, and improved vision plus systematic business mentoring could be the solution.

Without proper glasses, these women struggle with tasks at work and home, often relying on others and taking longer to finish things. But once they acquired glasses for close-up work, their vision and work comfort improved dramatically, leading to enhanced productivity. This improvement translated into higher earnings, financial stability, increased savings and reduced product rejections. The documented challenges of presbyopia and the benefits of addressing it are well established globally.^4 7 26 27^

Beyond work, the glasses improved their daily lives. They could now read, write and use phones easily, leading to greater independence, confidence and overall well-being. This mirrors a study among South African textile workers^28^ and Goertz *et al*’s review.^29^ Stronger social connections followed, as they became more active in their communities. This aligns with research showing health improvements strengthen social bonds and individual well-being.^30^ Improved vision likely boosted their confidence and social skills, making them valuable community assets. Helping others with similar issues further strengthened their sense of purpose and community ties.^31^

Initially, the craftswomen did not realise their vision problems were treatable. This, like Steinmetz *et al*’s findings,^32^ was a major barrier to seeking help. Lack of education and awareness programmes likely played a role, with limited access to information, cultural factors, and poor promotion of eye care services. Closing this knowledge gap is crucial. Educating craftswomen about their vision can encourage them to seek help, promoting better eye health overall. Getting glasses dramatically improved their vision and comfort, motivating them to wear them. This aligns with research showing positive experiences lead to behaviour change.^30^ Feeling better physically and more confident thanks to good vision creates a positive feedback loop, motivating them to take care of their eyes. This suggests health interventions should emphasise both physical and psychological benefits to promote proactive health behaviours.

However, a purely vision-centric approach, which focuses primarily on improving vision care and addressing vision-related issues, is insufficient to solve the intersectionality of older women entrepreneurs in Zanzibar. Improved vision leads to higher production, but limited market access and fierce competition force them to lower prices. This, in turn, necessitates compromising on quality, potentially limiting their ability to attract new customers, especially tourists. The NGOs are creating a situation where a select group of entrepreneurs have significant cost advantages, access to modern technology and easier access to capital. This makes it extremely difficult for the independent craftswomen, even with their improved productivity due to the glasses, to compete effectively.

This analysis of the Zanzibari craftswomen’s experiences sheds light on a complex issue: the limitations of improved vision without addressing broader economic and business challenges. While studies^4 7 26 27^ have documented the positive impact of improved vision on productivity in LMICs, the Zanzibari case highlights the need for a multifaceted approach.

The narrative reveals a restricted market for the craftswomen’s products, similar to findings by Donovan and Poole on limitations faced by female artisans in the Caribbean.^33^ This limited geographical reach, as the craftswomen themselves point out, fosters intense competition, a phenomenon observed among documented smallholder agriculture in developing economies.^34^ This competition drives down prices and incentivises the use of lower-quality materials, hindering product innovation, which is crucial for business growth.^35^

The situation is further complicated by the presence of international NGOs supporting other entrepreneurs. These NGOs, as the narrative suggests, create an uneven playing field by providing subsidised materials, equipment and access to capital. This advantage, similar to concerns raised by Edwards^36^ regarding aid dependency, allows the NGO-backed businesses to produce faster and potentially with a more modern aesthetic, further marginalising the independent craftswomen.

Beyond the immediate challenge of competition, the narrative underscores the craftswomen’s lack of formal business training. This lack of knowledge in areas like financial management, marketing and loan application processes mirrors findings by Arshed *et al*^37^ on the limitations faced by women entrepreneurs in emerging economies. Additionally, the digital divide, where some craftswomen struggle with social media marketing, is a hurdle also observed on gender and digital entrepreneurship in India.^38^ The proposed business mentoring programme directly addresses these skill gaps and offers a promising solution. Research on entrepreneurship training programmes in developing countries^39 40^ suggests that such programmes can equip individuals with the necessary skills to navigate challenges and achieve sustainable growth.

The limitations of using solicited diaries are acknowledged. These diaries may not capture the broader social, economic and cultural context influencing the diarists’ experiences, and it can be challenging to verify the accuracy of the information provided. Some entries were brief, particularly close to months 5 and 6. Additionally, diarists might hesitate to record negative experiences or portray themselves negatively, leading to potentially inaccurate or incomplete information. To address these limitations, we followed up with listening workshops to gain a more comprehensive understanding and shared interpretations of the narratives with the craftswomen to ensure accuracy. While the findings may not be applicable to a broader population beyond the specific group of older craftswomen, our aim is to provide them with a democratic platform to devise interventions that could further benefit them. Lastly, it was not possible to include craftswomen from different crafts requiring varying levels of near visual precision which may have influenced individual experiences of presbyopia correction.

This study offers insights into how improved near vision may support older Zanzibari craftswomen’s productivity, financial confidence and well-being. However, the findings underscore that vision correction alone is unlikely to address the intersecting challenges they face as older women entrepreneurs in a low-resource, patriarchal setting. These include restricted market access, fierce competition and limited business skills. A more holistic approach, combining vision care with tailored business mentoring and structural support, may be needed to respond meaningfully to the intersectionality shaping their experiences and opportunities.

## Supplementary material

10.1136/bmjopen-2024-090883 online supplemental file 1

## Data Availability

Data are available on reasonable request.
