# Supplementary material for: What is the Zanzibari craftswomen’s experience on eyeglass use, business challenges and solutions? Participatory action research using solicited diaries and listening workshop narratives
Source: BMJ Open. 2025 Jun 10;15(6):e090883. doi: 10.1136/bmjopen-2024-090883 (PMC12182027; doi:10.1136/bmjopen-2024-090883)
Supplement: online supplemental file 1 [file bmjopen-15-6-s001.docx]

Supplemental Table S1: Diary excerpts from the craftswomen

| Entry | Entry Identification Number | Diary excerpt |
| --- | --- | --- |
| 1 | A1 | I can't believe how much my life has changed since I started wearing glasses. The constant tearing has finally stopped, and my eyes no longer feel sore and irritated, as if there were grains of sand scratching them. It's such a relief! I can work without that unbearable discomfort. |
| 2 | F1 | Today, I feel such a relief. My eyes are no longer sore, swollen, or itchy. It's like a weight has been lifted off my shoulders. I can finally read, write, sort the rice, and even sew again without any discomfort. It's such a simple thing, but being able to see clearly and do these tasks has brought me so much joy. |
| 3 | J1 | Before I had reading glasses, I could not do my work properly; I could not thread the needle or sew. |
| 4 | B1 | Today was incredibly frustrating. My eye impairment has made even the simplest daily activities a struggle. Take making soap, for instance – something I usually enjoy. But today, my eyes wouldn't stop tearing up. It made the whole process so difficult. I just wish I could go through my day without this constant trouble. |
| 5 | J4 | I never imagined how life-changing reading glasses could be. Before I started using them, I struggled so much with my tasks. It was incredibly frustrating and took forever to get anything done. I remember trying to thread a needle or knit, and crochet – I wasted so much time. But now, with my reading glasses, everything is so much clearer and easier. I feel capable again. |
| 6 | F1 | Today, I found myself reflecting on the past and how I used to struggle with my work. Those days were tough. My near vision was so blurry. I remember the frustration, having to constantly ask others for help. It made me feel so dependent. But now, looking back, I see how far I've come and how much I've grown from those challenges. |
| 7 | B1 | Before I had my glasses, work was a constant struggle. Every task was challenging, and I found myself relying heavily on others for help. It was incredibly frustrating. Every time I tried to focus on my work, whether alone or in a group, my vision issues would get in the way. It was so disheartening to see my teammates bustling around, completing their soap making with ease, while I have to strain my eyes, desperately trying to keep up. I felt like such a burden. I questioned my abilities. |
| 8 | C3 | I can’t even begin to express how happy I am right now! My new glasses have made such a difference. Every day, I feel my vision getting better and better. Last month, I struggled so much with my work, but now I’m working through it comfortably and finishing tasks faster than ever. It's such a relief. |
| 9 | C2 | Today, I felt relief and happy as I realized just how much my new reading glasses have changed my life. Wearing them, I find myself working with faster, confidence, and comfort. It’s incredible how something so simple can make such a huge difference. Just last month, I completed sewing a mat that was 60 meters long. To my surprise, it only took me 2 months! Before I had these glasses, a project of that size would have dragged on for 3 months or more. It feels like a weight has been lifted off my shoulders, and I can’t help but feel grateful for my glasses. |
| 10 | A2 | What a month it has been. What remarkable improvement in my work quality. Thanks to my new eyeglasses. It's incredible how something so simple can make such a difference. This month has been particularly rewarding. I’ve seen a significant boost in my income. I started the month with a saving of TSZ75,000, and to my delight, it has grown steadily. Compared to my previous earnings, this increase feels like a breath of fresh air. Even more encouraging is the fact that I haven’t had a single case of rejected sales. The clarity and precision the eyeglasses provide have played a role in this success. Not only has my income improved, but I’ve also been able to save more than I expected.  I feel an overwhelming sense of gratitude for these changes. It’s amazing how a simple pair of glasses has not only improved my vision but also brought about a clearer, more prosperous future. |
| 11 | B1 | Today, there was talk of a trip or other activities planned, and I found it incredibly challenging to join because I can’t see well. The mere thought of having to participate just overwhelmed me. |
| 12 | D1 | Before I started wearing reading glasses, studying in Quran forums was such a struggle for me. I remember those days—the frustration of not being able to see clearly, of only listening while others were writing or reading from the board. My vision problems made it hard to keep up. I felt like I was always lagging behind, unable to complete tasks as required. |
| 13 | E2 | Today, I've come to truly appreciate the value of reading glasses. With them, I can accomplish my tasks promptly, finding satisfaction in my work. They allow me to see clearly, whether it's reading, writing, sewing—every detail is sharp, every word is clear. Even threading a needle or using my phone becomes effortless. It's amazing how something so simple can bring such immense benefits to my daily life. Today has been a reminder of how these little things can make a difference. |
| 14 | I1 | Today was a significant day for me. I found that wearing glasses has been incredibly helpful. They make my work so much easier, allowing me to focus and accomplish tasks with greater clarity and efficiency. In addition to my work, I actively participated in various working groups with my peers. Later, I spent some time at the madrasa, engaging in reading sessions. At home, I cherished the opportunity to assist my children with their studies. Seeing their progress and being a part of their education brings me immense joy and satisfaction. It's moments like these that remind me of the importance of family and the blessings of being able to support them in their growth. Today was a reminder of how fortunate I am to have the reading eyeglasses and opportunities that enable me to thrive. |
| 15 | J3 | Reading glasses have proven to be a blessing. They've made it possible for me to do my homework effortlessly. For instance, I can now pick grains of rice and trim my nails without any difficulty. It's such a relief to regain this independence! |
| 16 | E3 | Today, I feel a sense of happiness and confidence. It's amazing how much wearing glasses has changed things for me. I can now participate fully in activities that used to be challenging. It's a reminder of how important it is to seek help from others when we need it. I'm grateful for this change. |
| 17 | G1 | Today I felt terrible. My new glasses have helped me to work quicker, and allowed me to work comfortably, even finishing tasks ahead of schedule. But then, disaster struck. My glasses shattered. Suddenly, everything turned blur. I felt so uncomfortable, unable to see clearly. It's amazing how something so small can have such a big impact on your day. I hope I can get them fixed or replaced soon. Until then, I'll have to navigate. |
| 18 | D2 | Today has been such a fulfilling day. I find immense joy in my work, especially with the aid of my eyeglasses. They enable me to utilize my skills effectively, resulting in the creation of high-quality products. What's even more gratifying is knowing that through my work, I can support fellow women entrepreneurs. It's not just about what I accomplish for myself; it's about the impact I can have on others' lives too. |
| 19 | B3 | Today has been such a fulfilling day in our community. The connection I've built with fellow craftswomen and others because of wearing glasses has been incredibly positive. People are genuinely interested in how they can get glasses for themselves, especially those with eye problems. It's heartening to see how wearing glasses not only helps me but also sparks conversations and empathy among us. Moreover, I've noticed other craftswomen seeking our advice on improving their products, inspired by the difference glasses have made in my work and life. It's a wonderful feeling to be able to share knowledge and support each other in our crafts. These moments remind me of the power of community and how something as simple as wearing glasses can bring us closer together, helping us all grow and thrive. |
| 20 | C4 | Today marks a turning point in my life. I've managed to rebuild positive relationships with my relatives, community members, and family. Through hard work and determination in my job, I've started contributing to our household expenses. Now, I can proudly support our daily needs such as buying food and paying school fees for the children. I no longer depend on others for assistance in my work. It's amazing to see how far I've come, and I'm grateful for the support and opportunities that have led me to this moment. |
| 21 | A1 | Before I got glasses, I felt lost and helpless when it came to dealing with my eye problems. The thought of going to the hospital for tests was daunting; I had no clue about what to expect or how to handle it. I convinced myself that my vision issues were permanent, something I'd have to live with forever. It was frustrating, not knowing there could be a solution out there for me. |
| 22 | G1 | Today, I met some people struggling with vision problems, and it really struck a chord with me. It's heartbreaking to see individuals who haven't received encouragement to correct their vision. It feels like they've been overlooked, left to navigate the world with this challenge alone. It made me realize how important support and guidance can be in overcoming such obstacles. |
| 23 | A1 | I felt happy as I was able to motivate those who were struggling to find hope. Seeing their faces light up brought tears to my eyes. It's moments like these that remind me why I want to help and inspire others. I am filled with gratitude for the opportunity to make a positive impact, and I hope this feeling stays with me for a long time to come. |
| 24 | C1 | I feel like so many others are facing the same challenges as me. It's this overwhelming need for awareness and encouragement to correct our vision. It's not just about seeing clearly, but also about goals and aspirations. Sometimes, it feels like we're all facing all these problems together, searching to where we want to be. I hope we can all find the strength and support we need to keep pushing forward, despite the obstacles in our way. |
