# Supplementary material for: What is the Zanzibari craftswomen’s experience on eyeglass use, business challenges and solutions? Participatory action research using solicited diaries and listening workshop narratives
Source: BMJ Open. 2025 Jun 10;15(6):e090883. doi: 10.1136/bmjopen-2024-090883 (PMC12182027; doi:10.1136/bmjopen-2024-090883)
Supplement: Supplementary file 2 [file bmjopen-2024-090883.reviewer_comments.pdf]

PEER REVIEW HISTORY

BMJ Open publishes all reviews undertaken for accepted manuscripts. Reviewers are asked to complete a checklist review form and are provided with free text boxes to elaborate on their assessment. These free text comments are reproduced below.

ARTICLE DETAILS

Title (Provisional)

What is the Zanzibari Craftswomen's Experience on Eyeglass Use, Business Challenges, and Solutions?: Participatory Action Research using Solicited Diaries and Listening Workshop Narrative

Authors

Omar, Fatma; Pillai, Bhagyalaxmi Shivalingam; Othman, Omar Juma; Holland, Eleanor; Mashayo, Eden; Shah, Kajal; Graham, Ronnie; Graham, Christine; Chan, Ving Fai

---

VERSION 1 - REVIEW

**Reviewer**	**1**
**Name**	**Sandi, Frank**
**Affiliation**	**University of Dodoma School of Medicine and Nursing, Ophthalmology**
**Date**	**17-Mar-2025**
**COI**	**None**

The paper adds a lot in innovative measures of dealing with refractive errors especially in forgotten group. Few edits:

1. Line 139, mentions 10 women cooperatives but in Table 1 it is 8 groups seen

2. Line 143, says one woman from each cooperation was selected but table 1 shows there are 2 women from tailoring and weaving cooperatives. Also there isno explanation of how the 10 women were selected.

3. Line 161, how were the 20 women selected?

4. Table 2, why only 24 Diary excerpts from the craftswomen and not from all 49 entries?

5. How did the authors account for different types of crafts activities requiring different visual acuity levels?

6. Line 500, the reference has no year of publication.

**Reviewer**	**2**
**Name**	**de la Perrelle, Lenore**
**Affiliation**	**Flinders University, of Education, Psychology and Social Work**
**Date**	**27-Mar-2025**
**COI**	**None**

Thank you for the opportunity to review this interesting manuscript. The intersectionality of low vision, independent craft workers and market context is useful to address the complexity of work equity in LMICs.

I have several minor comments and methodology suggestions to strengthen the manuscript.

1. use consistent descriptions Eyeglass vs spectacle correction and near reading eyeglasses in abstract, and spectacle in Keywords. if one term is preferred in country then say that.

2. L 61 in Introduction introduce the condition in plain language such as 'common age related loss of near sight, known as presbyopia' will be more clear to non Vision specialists.

3. L77 a definition and reference to PAR is needed and how quality is assured. (Ozano et al 2020)

It is useful to identify earlier that this study presents findings from stage 5 only for clarity.

4. The methods section needs clarity.

add an overview of PAR and why it suits the project or required in LMIC research,

then data collection methods, diaries and listening workshops why these two steps were chosen.

Then data analysis: basis of a narrative analysis, interpreting stories, thoughts, feelings and experiences, hermeneutics and the coding approach used.

this will strengthen the theoretical underpinnings of this study.

5. Results: table 2 is long and while it adds significant insight into the experiences, it may be summarised or added as a supplementary file.

6. Check the consistent use of tense in discussion such as at l312 got/ get glasses then comfort improved/ improves.

VERSION 1 - AUTHOR RESPONSE

1. Line 139 mentions 10 women cooperatives but in Table 1 it is 8 groups seen.

Response: We appreciate this observation. The discrepancy arose because while 10 diarists were selected, their crafts fell under 8 broad cooperative categories. We have now revised the manuscript to clarify that participants came from 8 distinct cooperative types, with 10 diarists selected overall.

Change Made:

“The study focused on 10 diarists from 8 women’s cooperatives, representing different craft activities…”

1. Line 143 says one woman from each cooperative was selected but Table 1 shows 2 women from tailoring and weaving cooperatives. Also, there is no explanation of how the 10 women were selected.

Response: Thank you for pointing this out. We have clarified that in tailoring and weaving cooperatives with larger memberships, two women were selected to ensure broader representation. The selection process—based on diagnosis of presbyopia and writing proficiency—is now explained more clearly.

Change Made:

“One woman from each cooperative was initially selected; however, in tailoring and weaving cooperatives with larger memberships, two were chosen to enhance diversity. Selection was based on diagnosis of presbyopia and ability to maintain written diary entries.”

2. Line 161 – how were the 20 women selected for the listening workshop?

Response: We thank the reviewer for this helpful suggestion. We have clarified in the Methods section that women were purposively sampled to ensure diversity in age, geographic location, and craft type from among those who received eyeglasses.

Change Made:

Added to Methods:

“Participants in the listening workshops (n=20) were purposively selected from among those who received eyeglasses, ensuring representation by age, geography, and craft type.”

3. Table 2: Why only 24 diary excerpts from the craftswomen and not from all 49 entries?

Response: We appreciate this question. We selected 24 diary excerpts based on thematic richness and their illustrative power within the analysis. To maintain transparency, we have stated that all 49 entries are available on request as they contained intimate, confidential and identifiable data.

Change Made:

Clarified in Results:

“Twenty-four excerpts were selected for their thematic richness and relevance. All 49 diary entries could be obtained on request as they contained intimate, confidential and identifiable data.”

4. How did the authors account for different types of crafts activities requiring different visual acuity levels?

Response: Thank you for this important point. We have added a statement in the Limitation to acknowledge that crafts vary in their visual demands and this may have influenced how presbyopia correction impacted individuals.

Change Made:

Added to Limitation:

“Different crafts required varying levels of near visual precision, which may have influenced the degree of impact experienced by each participant.”

5. Line 500, the reference has no year of publication.

Response: Thank you. We have corrected the reference.

6. Use consistent descriptions: eyeglasses vs. spectacles. In abstract use of “near reading eyeglasses,” “spectacle correction,” and “spectacle” in keywords.

Response: We agree and have standardized the term “eyeglasses” and “eyeglass” throughout the manuscript, including the abstract and keywords. Exceptions were made only when citing original references that used “spectacles.”

7. Line 61 in Introduction – introduce the condition in plain language.

Response: We have revised the sentence for clarity and accessibility to a non-specialist audience.

Change Made:

“Presbyopia, a common age-related condition that affects the ability to see clearly at close distances, hampers near-vision tasks.”

8. Line 77 – a definition and reference to PAR is needed and how quality is assured (e.g., Ozano et al. 2020). Also useful to identify earlier that this study presents findings from Stage 5 only.

Response: Thank you for this helpful suggestion. We have added a definition of PAR and cited Ozano et al. and Baum et al., as how quality assurance was maintained. We also clarify that the paper presents only Phase 5 findings.

9. The Methods section needs more clarity. Please add:

- An overview of PAR and its relevance in LMICs.
- Why solicited diaries and listening workshops were chosen.
- Details on narrative analysis and coding approach.

Response: We thank the reviewer for this excellent suggestion. We have revised the Methods section to include:

a) An overview of PAR and its use in LMIC contexts, as per Chevalier and Buckles.
b) Justification for using diaries and workshops, as per Jacelon et al., McGregor and Thomas and Znaniekci.
c) Explanation of our narrative analysis process as per Sparke’s, including reference to Riessman’s framework.

10. Table 2 is long. Consider summarizing or moving it to a supplementary file.

Response: We agree and have moved Table 2 to the Supplementary Table S1 to streamline the main text.

11. Check consistent use of tense in discussion, e.g., ‘got/get glasses’ and ‘comfort improved/improves’.

Response: Thank you. We have revised the discussion section to ensure consistency in verb tense, using past tense to describe study findings and present tense for general implications.

We hope these revisions address all concerns satisfactorily. Thank you again for the opportunity to improve our manuscript.

VERSION 2 - REVIEW

**Reviewer**	**1**
**Name**	**Sandi, Frank**
**Affiliation**	**University of Dodoma School of Medicine and Nursing, Ophthalmology**
**Date**	**21-Apr-2025**
**COI**	

The authors have addressed most of the queries and suggestions raised previously, but this time, they have not included a Methods section in the abstract. They just wrote the subtitle Results, but there is no content.

There is a subtitle for design, but I'm not sure if that's what they are referring to as Methods. They need to look at this and rectify it.
